# *In vitro* RNA-seq-based toxicogenomics assessment shows reduced biological effect of tobacco heating products when compared to cigarette smoke

**DOI:** 10.1038/s41598-018-19627-0

**Published:** 2018-02-05

**Authors:** Linsey E. Haswell, Sarah Corke, Ivan Verrastro, Andrew Baxter, Anisha Banerjee, Jason Adamson, Tomasz Jaunky, Christopher Proctor, Marianna Gaça, Emmanuel Minet

**Affiliations:** British American Tobacco R&D Centre, Regents Park Road, Southampton, SO15 8TL UK

## Abstract

The battery of regulatory tests used to evaluate the risk of novel tobacco products such as heated tobacco products (THPs) presents some limitations including a bias towards the apical endpoint tested, and limited information on the mode of action. This is driving a paradigm shift to more holistic systems biology approaches. In this study, we used RNA-sequencing to compare the transcriptomic perturbations following acute exposure of a 3D airway tissue to the aerosols from two commercial THPs and a reference 3R4F cigarette. 2809 RNAs were differentially expressed for the 3R4F treatment and 115 and 2 RNAs for the two THPs (pFDR < 0.05, FC > 1.5), respectively. The relationship between the identified RNA features and gene ontologies were mapped showing a strong association with stress response, xenobiotics metabolism, and COPD-related terms for 3R4F. In contrast, fewer ontologies were found enriched for the THPs aerosols. “Response to wounding” was a common COPD-related term over-represented for the two THPs but at a reduced significance. Quantification of a cytokine panel post-exposure confirmed a pro-inflammatory effect of cigarette smoke but not for THPs. In conclusion, THPs have a reduced impact on gene expression compared to 3R4F.

## Introduction

The health risk associated with smoking cigarettes and tobacco regulations have been driving a wave of development and innovation in next generation tobacco and nicotine delivery systems^1^. Such innovations include electronic cigarettes and heated tobacco products. Tobacco heated devices are distinct from combustible cigarettes and use resistance heating to generate an aerosol directly from a tobacco rod rather than burning tobacco^2,3^. This generates a simpler aerosol containing lower levels of smoke related toxicants^2,4,5^ and potentially offering a safer alternative to combustible cigarettes^1^.

Due to their recent introduction and availability limited to a few countries, there is an absence of any epidemiological data to support the potential reduced risk associated with the use of resistance heated tobacco products. Given the rapid development of THPs, there is a requirement to assess and understand how they compare to conventional tobacco smoking and a sham air control. One way of assessing the relative safety of these products is to investigate the toxicological profile of THP aerosols using *in vitro* testing, as part of a weight-of-evidence approach.

Traditional *in vitro* genotoxicity testing adheres to international guidelines and these batteries of assays are required by regulatory agencies as part of a product safety assessment approach. For example, the International Conference on Harmonisation^6^ and the Cooperation Centre for Scientific Research Relative to Tobacco^7^ recommend the use of a bacterial mutagenicity assay (Ames reverse mutation assay); a mammalian cell based assay for cytogenetics/mutation, (*in vitro* micronucleus); chromosome aberrations or the mouse lymphoma assay (MLA) and a cytotoxicity based assay. We have recently demonstrated significantly reduced *in vitro* mutagenicity and cytotoxicity of THPs when compared to reference 3R4F conventional cigarettes^8,9^. Such tests, however, present some limitations including uncertainties regarding bacteria/animal to human cross-species predictivity, bias toward the apical endpoint tested, and none or only partial information on the mode of action of any given chemical^10^. Recognizing these limitations there has been a shift by the toxicological community towards more holistic alternative systems biology approaches. The National Research Council’s “Toxicity Testing in the 21st Century” paradigm outlines the utility of such approaches using human tissues, high throughput profiling screens and ‘omics to dissect the cellular-response networks to support the mechanistic understanding of toxicity pathways^11^. For instance, Moffat and colleagues have compared the predictivity of toxicogenomics and traditional genotoxicity assays using the carcinogen B[a]P as test compound and concluded that both were predictive of DNA damage^10^. Toxicogenomics, however, offered additional information on the genotoxic and non-genotoxic mode of action of B[a]P^10^.

We and others have previously used a systems biology approach to demonstrate the reduced effect of electronic cigarette (e-cigarette) vapor compared to smoke from a reference cigarette using RNA-seq and microarrays^12–14^. Two of those studies also reported some effect on metabolic/biosynthetic processes, apoptosis, hypoxia and extracellular membrane pathways after an acute exposure to e-cigarette vapor compared to air^13,14^. This demonstrated the value of a transcriptomics screen in detecting functional changes that are not typically assessed through classic regulatory toxicology assays. Yet the expression of many genes was only marginally altered by e-cigarette vapor and therefore additional high density/sensitive functional screens would add value to the gene expression data to confirm the cellular impact.

RNA microarray-based systems biology studies have also been used *in vitro* and *in vivo* to assess the adverse effect of one commercial heated tobacco system (THS) using either puff or nicotine as a dose reference^15,16^. The data demonstrated a reduced impact of heated tobacco aerosol on differential gene expression compared to smoke from a reference cigarette^15,16^. These observations were in line with the reduction of toxicants measured in the aerosol of the heated tobacco product^5^. A variety of heated tobacco devices are now available, each with different designs and operating temperature leading potentially to different aerosol chemistries. Hence it is not known to what extent different heated tobacco devices lead to different biological responses and toxicogenomic profiles.

**Figure 1:**
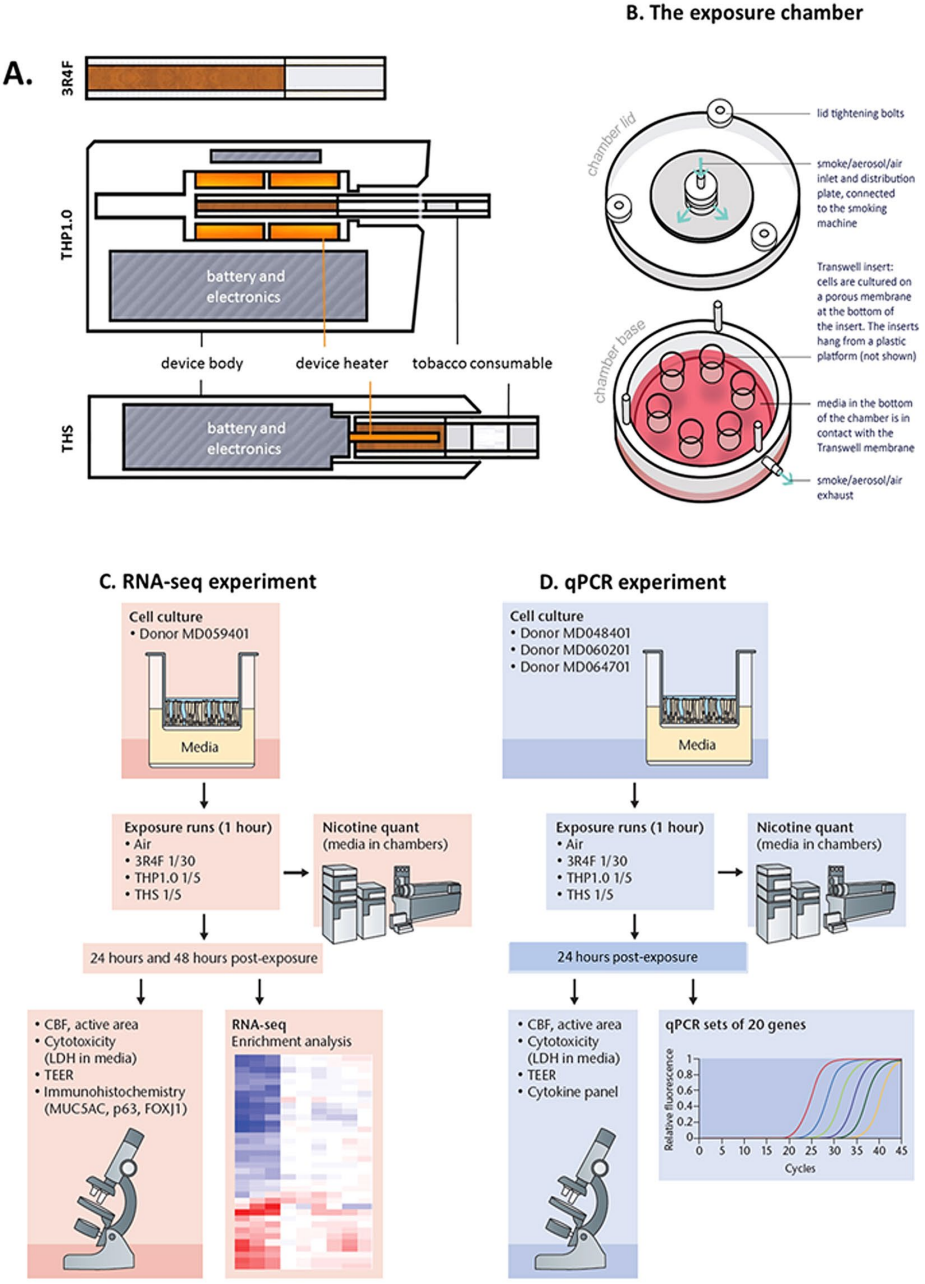
Test product schematics and experimental design summary. (**A**) Cross section schematic representation of the 3 test products used in this study: 3R4F reference combustible cigarette; THP1.0 having a slim size tobacco consumable inserted in the heating device with the peripheral heating components and THS having a king size tobacco consumable with a centrally positioned heating blade. (**B**) Aerosol exposure chambers with the cell inserts position (Figure adapted from Haswell *et al*.^13^). (**C**) RNA-seq experimental design. MucilAir^TM^ (donor MD059401) reconstituted airway tissues were exposed to aerosols from 3R4F, THP1.0, THS and to air at the indicated dilutions (Figure adapted from Haswell *et al*.^13^). The media in the exposure chambers were collected for nicotine quantification at the end of the exposure. Following 24 and 48 hrs post-exposure, MucilAir^TM^ tissues were assessed for functional endpoints and RNA isolated for RNA-seq. A minimum of 3 independent repeats were conducted with 3 cell inserts per treatment. (**D**) Validation of results in multiple MucilAir^TM^ donors by qPCR (Figure adapted from Haswell *et al*.^13^). Three different donors (MD048401, MD060201, MD064701) were exposed to aerosols of 3R4F, THP1.0, THS at the shown dilutions, and to air. Three technical replicates were used per donor and treatment. Nicotine was quantified as previously described. Functional endpoints were assessed in MucilAir^TM^ and supernatants collected for cytokine quantification and total RNA extracted for qPCR at 24 hrs post-exposure.

Since THPs are a new tobacco product category, our objective was to conduct an *in vitro* toxicogenomics-based assessment of two commercial THPs (THS and THP1.0) with different designs (Fig. 1A) and compare them to a reference 3R4F conventional tobacco product. The THS product uses a king size tobacco consumable heated at its center by a blade (Fig. 1A)^3^, the THP1.0 uses a slim tobacco consumable heated peripherally^17^ (Fig. 1A). *In vitro* exposure of commercially available primary 3D airway cultures (MucilAir™) to THP aerosols were conducted at the air liquid interface (ALI) using nicotine as a dose surrogate and an aerosol dilution aiming to exceed the nicotine delivered by 3R4F. The gene expression profiling was performed using RNA-seq on MucilAir™ samples at 24 and 48 hrs post-exposure to either air, aerosol from the two THPs, and 3R4F reference cigarette (Fig. 1A). The exposure runs were conducted using a standardized intense smoking regimen on a Borgwaldt RM20S smoking engine, as previously described^13^. A common aerosol dilution was used for the two THPs that exceeds the nicotine delivered by the 3R4F reference cigarette at same puff number. Differential gene expression analysis combined with functional and morphological endpoints were performed to assess the cellular response to the aerosols from the different products.

## Results

### Dosimetry

**Figure 2:**
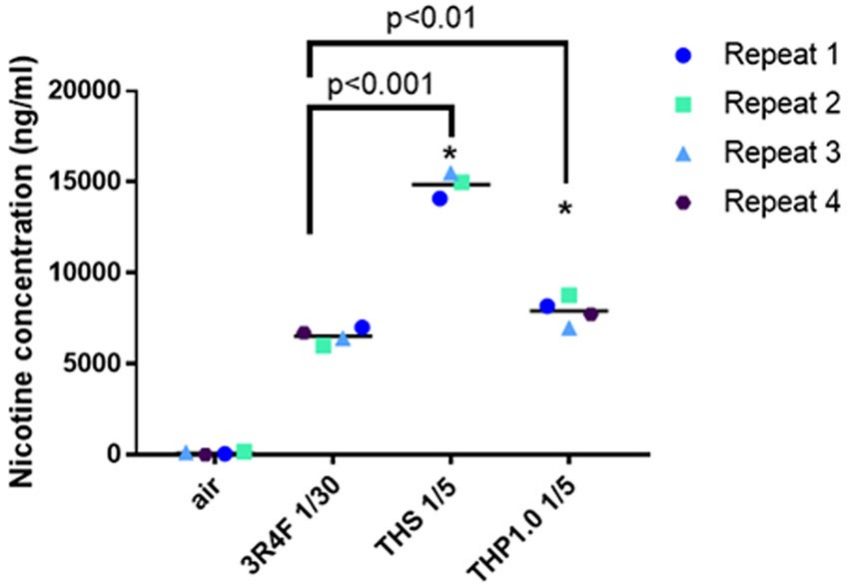
Nicotine quantification in the cellular media from exposure chambers. Scatter plot showing the nicotine concentration (ng/ml) quantified for each experimental repeat and per treatment. The values from independent run repeats are labelled with different colors. The mean nicotine value for each treatment is shown by the black horizontal line. *Denotes a t-test significance at p < 0.05 using 3R4F nicotine as comparator.

Reconstituted 3D human airway cells (MucilAir™) from one donor (MD059401) were exposed at the ALI to reference 3R4F cigarette smoke, THP1.0, and THS aerosols for an hour using a Borgwaldt RM20S smoking engine. Figure 1A provides a schematic of the products used in the study, Fig. 1B (Figure adapted from Haswell *et al*.^13^) represents an exposure chamber, and a summary of the transcriptomics study design is shown in Fig. 1C (Figure adapted from Haswell *et al*.^13^). For the RNA-seq studies, 3 independent exposure runs were performed each with 3 technical replicates totaling 9 cell inserts in total per treatment and time point. A 4^th^ run was performed to replace samples damaged in the previous runs. As described previously^13^, nicotine was quantified in the cellular media from the exposure chambers to assess aerosol dose. Using a Borgwaldt RM20S smoking engine to generate aerosols, in this study a smoke dilution of 1/30 was used for 3R4F cigarette smoke corresponding to the approximate dilution of smoke in human airways^18^. For the THPs, our objective was to use a more concentrated aerosol with nicotine in excess of 3R4F nicotine as a dose reference. We used a 1/5 dilution to generate aerosols for both THP1.0 and THS, to exceed nicotine delivery compared to 3R4F. The THS and the nicotine values are reported in Fig. 2. At the dilutions tested 6528 ± 431.6 ng/ml nicotine was measured in the media of Mucilair™ exposed to 3R4F smoke compared to the air control of 99.3 ± 72.6 ng/ml (Fig. 2). THS delivered approximately twice as much nicotine, 14866.7 ± 709.5 ng/ml, p < 0.001 compared to 3R4F and the THP1.0 product delivered 7912.5 ± 763.6 ng/ml significantly higher (p < 0.01) than 3R4F (Fig. 2).

### Airway epithelia functional markers

The integrity of the reconstituted airway tissue from donor MD059401 was assessed at 24 hrs and 48 hrs post-exposure to 3R4F smoke, THS, and THP1.0 aerosols using a series of endpoints including: (i) cilia beat frequency (CBF), (ii) TEER for epithelium tight junction integrity, (iii) quantitative immunohistochemistry using FOXJ1 (marker of ciliated epithelial cells), MUC5AC (marker of goblet cells), p63 (marker of basal epithelial cells) to identify cellular sub-populations, and (iv) cell viability by LDH release.

**Figure 3:**
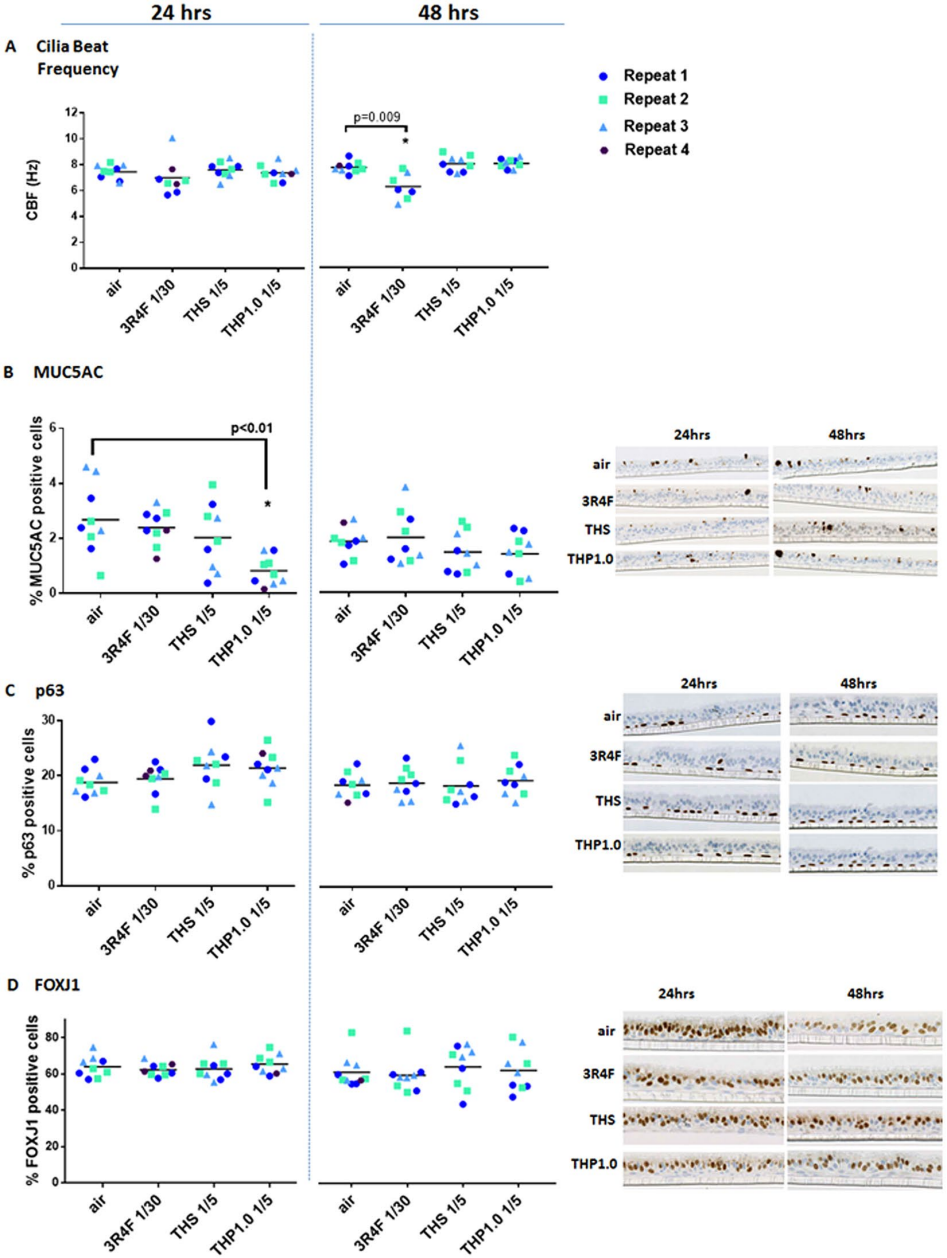
MucilAir™ functional and morphological endpoints after exposure to air, 3R4F smoke, THP1.0 and THS aerosols. Cilia beat frequency (Hz) scatter plots at 24 hrs and 48 hrs post-treatment (**A**). Scatter plots for MUC5AC (**B**), p63 (**C**) and FOXJ1 (**D**) quantitative immunohistochemistry at 24 and 48 hrs post exposure. Values from each independent exposure repeat are labelled with a different color. The mean CBF, MUC5AC, p63 and FOXJ1 values for each treatment is shown by the black horizontal line. *Denotes a t-test significance at p < 0.05 using the air control as comparator. Representative micrographs are shown in (**B**, **C** and **D**) for the MUC5AC, p63, and FOXJ1 immunohistochemistry, respectively.
(ii) A drop of TEER was observed in the cells treated with 3R4F smoke and was significant at 48 hrs (1384.2 ± 413.4 Ω) compared to the air control (1814.7 ± 202.9 Ω) (p < 0.05) (Supplementary Figure S2). Except for one MucilAir™ insert in the 3R4F treatment group 24hrs post-exposure, all inserts kept a TEER above 500 Ω, a value often considered as benchmark for tight junction integrity^21^. The TEER was not changed at 24 hrs and 48 hrs after an acute exposure to the THS and THP1.0 aerosols (Supplementary Figure S2).
(iii) Quantitative immunohistochemistry detected MUC5AC (goblet cells) (2.7 ± 1.3 % at 24 hrs; 1.9 ± 0.5 % at 48 hrs), p63 (basal cells) (18.8 ± 2.2 % at 24 hrs; 19.4 ± 2.3% at 48 hrs), and FOXJ1 (ciliated cells) (63.9 ± 5.8 % at 24 hrs; 62.4 ± 8.8 % at 48 hrs) in the air control (Fig. 3B to D). Overall, there was no significant changes for these markers following 3R4F, THS and THP1.0 exposures at all time points compared to the air control except for MUC5AC at 24 hrs post-exposure to the THP1.0 (0.8% ± 0.5 %) aerosol (p < 0.05) (Fig. 3B).
(iv) On average the LDH release was >5% for the different treatments and time points (Supplementary Figure S3).

(i) CBF, a marker of ciliated cell function^19^, appeared mostly constant across treatments and time points (Fig. 3A). A significant drop of CBF (6.2 ± 1.0 hz) was observed, however, 48 hrs after treatment with 3R4F smoke compared to air control (7.69 ± 0.43 hz). This was paralleled by a drop in the % of active ciliated cells at the surface of the inserts from 65.08 ± 12.6 % with air to 36.53 ± 29.45 % with 3R4F smoke, possibly indicative of cilia shedding^20^ (Supplementary Figure S1).

### Differential gene expression

**Figure 4:**
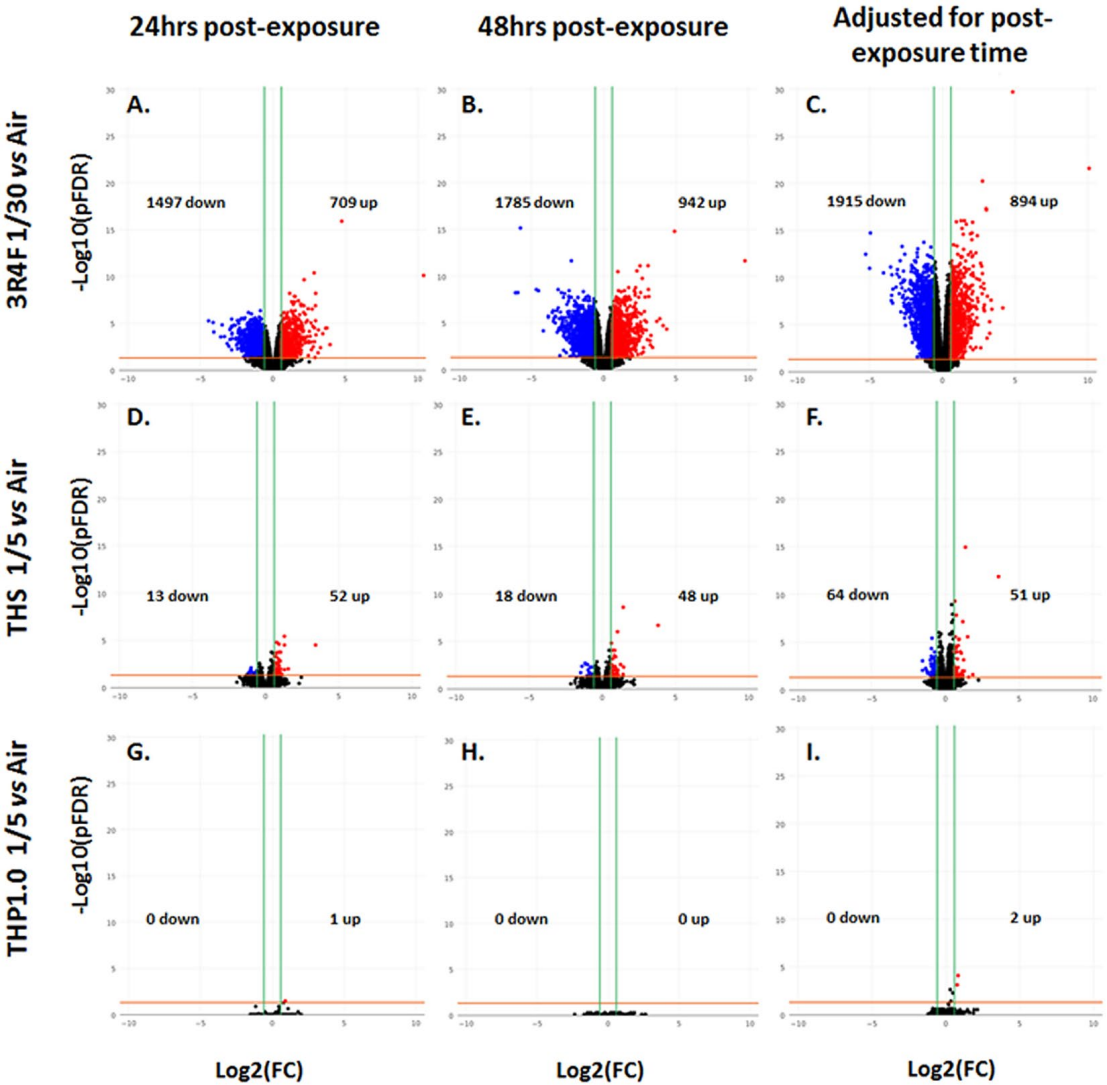
Volcano plots for the following 9 RNA-seq contrasts  and RNAs significant at [FC] > 1.5, pFDR < 0.05: 3R4F smoke exposure *vs* the air control presented in the top 3 volcano plots presenting the differential expression at 24 hrs post-exposure (**A**) the differential expression at 48 hrs post-exposure (**B**) and the differential expression adjusted for post-exposure time (**C**). The center volcano plots correspond to THS aerosol exposure *vs* the air control presenting the differential expression at 24 hrs post-exposure (**D**), the differential expression at 48 hrs post-exposure (**E**) and the differential expression adjusted for post-exposure time (**F**). The bottom volcano plots show the THP1.0 aerosol exposure *vs* the air control with the differential expression at 24 hrs post-exposure (**G**) the differential expression at 48 hrs post-exposure (**H**), and the differential expression adjusted for post-exposure time (**I**).

RNA-seq was applied to RNA samples extracted from MucilAir^TM^ at 24 and 48 hrs following 1 hour exposure to products (72 samples in total). All the samples passed RNA QC assessment, having a RIN above 8.0, however 5 samples failed the RNA-seq QC, and included three technical replicates distributed across two 3R4F exposures (24 hrs post-exposure time point), one THP1.0 (24 hrs post-exposure), and one air control (48 hrs post-exposure) (Supplementary Table S1). These 5 samples were excluded from the statistical analysis. The sequencing datasets (raw sequence files can be found at: https://www.ncbi.nlm.nih.gov/sra/SRP126155) were aligned to the human reference genome GRCh38^22^ and 22036 unique RNA features were mapped. The normalized RNA data from the test treatments (3R4F, THS, THP1.0) were analyzed *vs* the air control over a total of 6 contrasts (3R4F *vs* air, THS *vs* air, THP1.0 *vs* air at 24 and 48 hrs post-exposure). A further three contrasts were performed by combining the data from the 24 and 48 hrs time points to increase statistical power and extract as much information from the data as possible (3R4F *vs* air, THS *vs* air, THP1.0, adjusted for time). The differential expression results for these 9 contrasts are summarized in the volcano plots presented in Fig. 4 and Supplementary Table S1. At a pFDR < 0.05 and [FC (fold change)] > 1.5 the strongest differential expression response was observed for 3R4F exposure with 2806 RNA features at 24 hrs and 2727 RNA features at 48 hrs (Fig. 4A and B). Using the same thresholds, 65 RNA features at 24 hrs and 66 RNA features at 48 hrs responded to the THS aerosol exposure (Fig. 4D and E). Only one gene, EDN2 (endothelin 2), was found up-regulated at 24 hrs following exposure to the THP1.0 aerosol (Fig. 4G). When the data is adjusted for time 115 RNA features were identified for THS at pFDR < 0.05 and [FC] > 1.5 and 2 RNA features for THP1.0 (Fig. 4F and I). Up to 71 RNA features were differentially expressed for THP1.0 when using a higher pFDR threshold of 0.3 with no fold change filter (Supplementary Table S1). The full list of differentially expressed genes for the contrasts cited above is given in Supplementary Tables S2 to S12. More stringent fold change thresholds reduced the number of differentially expressed genes across all treatments. Between 121 and 226 genes were differentially expressed with fold changes greater than 4 at 24 hrs and 48 hrs post exposure for 3R4F smoke (pFDR < 0.01), respectively (Supplementary Table S1). Only CYP1A1 was induced by more than 4 folds after treatment with the THS aerosol (Supplementary Table S1).

### Enrichment analysis

**Figure 5:**
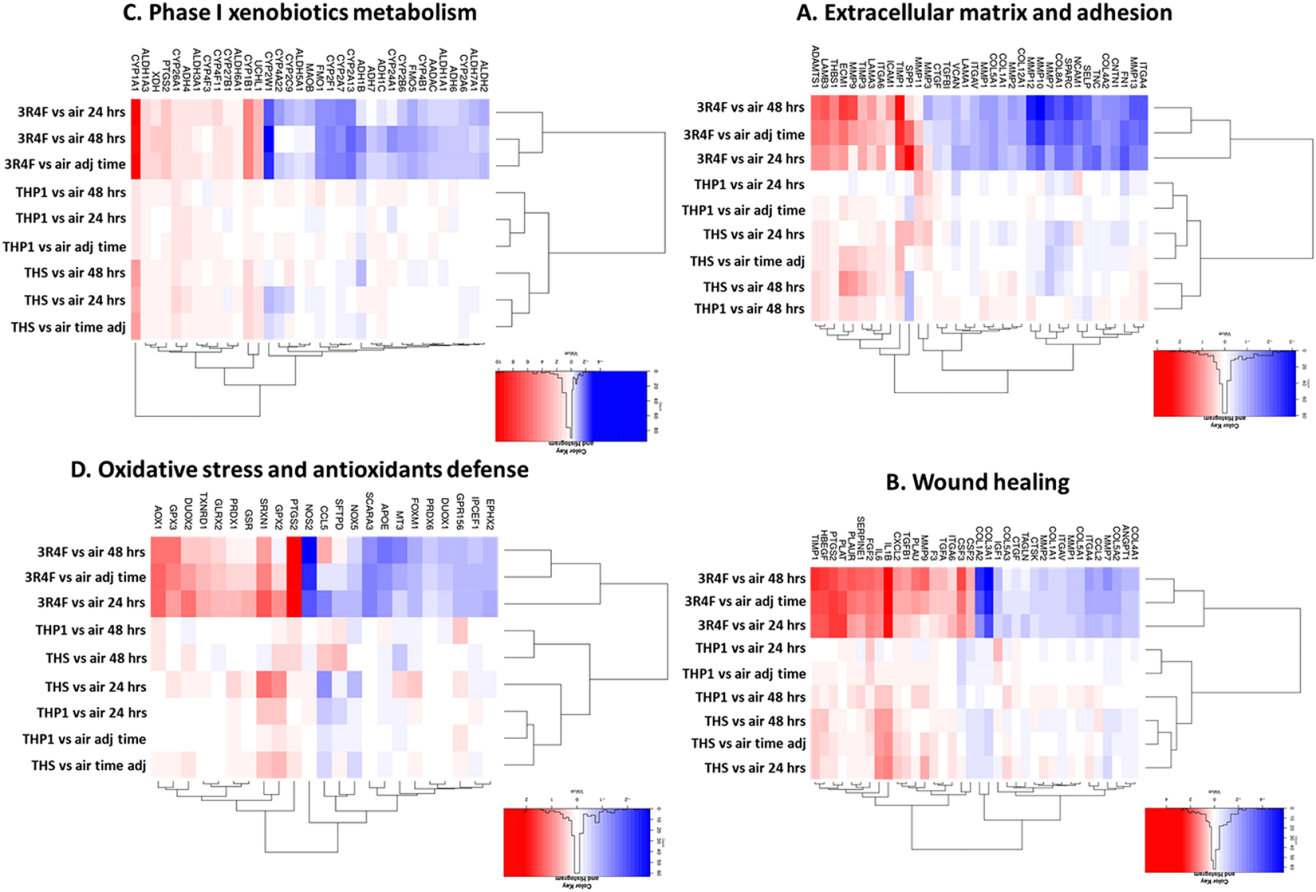
Unsupervised hierarchical clusters of genesets for extracellular matrix and adhesion (**A**) wound healing (**B**) phase I xenobiotic metabolism (**C**) oxidative stress and antioxidant response (**D**). Significant genes at pFDR < 0.05 and [FC] > 1.5 in at least one of the treatment. Contrasts are shown on the heat map with the log2(fold change) scale intensity. Blue indicates down-regulation and red indicates up-regulation.

Enrichment analysis were conducted to identify the functional pathways perturbed by the exposure to 3R4F smoke and the THS and THP1.0 aerosols. Two types of analyses were performed:

An unsupervised hierarchical clustering using 131 pre-defined pathway-focused genesets^23^ applied to the 9 contrasts for genes significant at pFDR < 0.05 and [FC] > 1.5 in at least one treatment. Examples of the top genesets significantly enriched (based on lowest pFDR value) included genes associated with “inflammatory response” (pFDR = 4.5e^−23^), “angiogenic growth factors” (pFDR = 2.2e^−17^), “wound healing” (pFDR = 1.0e^−14^), “extracellular matrix adhesion” (pFDR = 1.5e^−14^), and “drug/xenobiotic metabolism” (pFDR = 4.5e^−14^) (Fig. 5). Oxidative stress response is also shown in Fig. 5 since cigarette smoke is a known source of free radicals^24^. All four corresponding heatmaps are illustrated in Fig. 5 and the full dataset for the 131 genesets is provided in Supplementary Table S13. In the example genesets shown in Fig. 5, 3R4F treatment formed one cluster based on differential gene expression, while both THPs formed a second cluster. Distinct grouping for THS and THP1.0 was also observed in some genesets (B and C), for example “wound healing” (Fig. 5B) and “phase I drug metabolism” (Fig. 5C). This was mostly driven by upregulated genes such as CYP1A1, CYP1B1, CYP26A1, IL6, and IL1B with fold changes from 10 to 1.6, and downregulation of COL3A1 by 1.8-fold in the THS aerosol treatment (Fig. 5B and C, Supplementary Table S13). Gene markers of oxidative stress included induction of cyclooxygenase-2 (PTGS2), glutathione peroxidases 2 and 3 (GPX2, 3) and a strong downregulation of the nitric oxide synthase 2 (NOS2) by 3R4F smoke (Fig. 5D).

**Figure 6:**
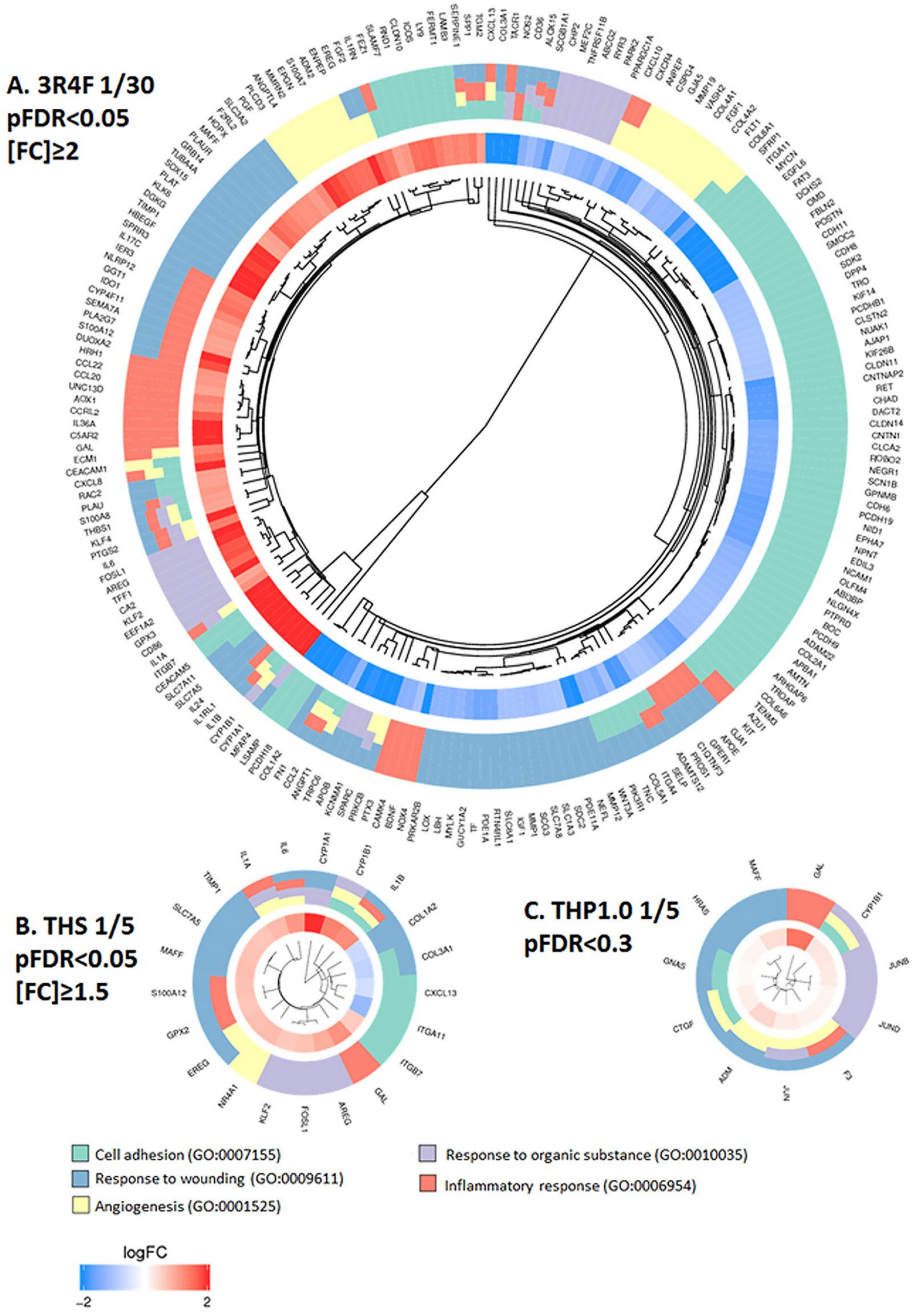
Cluster plots for differentially expressed RNAs matching with COPD-associated GO terms after treatment with 3R4F smoke and both THP aerosols. The genes that mapped to the GO terms are shown on the periphery of the plots. The inner circle shows the log2(fold change) for each gene, blue indicating down-regulation, red indicating up-regulation. The outer circle represents the GO term(s) assigned to the genes. The GO terms color legend and GO ID numbers are given at the bottom of the figure. (**A**) Cluster plot for differentially expressed genes matching with COPD-associated GO terms after treatment with 3R4F smoke (pFDR < 0.05, [FC] > 2, adjusted for time). (**B**) Cluster plot for differentially expressed genes matching with COPD-associated GO terms after treatment with THP1.0 aerosol (pFDR < 0.3, adjusted for time). (**C**) Cluster plot for differentially expressed genes matching with COPD-associated GO terms after treatment with THS aerosol (pFDR < 0.05, [FC] > 1.5, adjusted for time).

An untargeted gene ontology (GO) enrichment for biological process^25^ and a targeted GO cluster analysis for terms that have been associated with COPD^26^, was also performed. To have sufficient gene candidates to conduct this analysis, the following thresholds were applied to the time-adjusted contrasts: 3R4F *vs* air at pFDR < 0.05, [FC] > 2 (Supplementary Tables S1 and S5); THS *vs* air at pFDR < 0.05, [FC] > 1.5 (Supplementary Tables S1 and S8), and THP1.0 *vs* air at pFDR < 0.3 (Supplementary Tables S1 and S12). The enrichment results for the over-represented GO biological process terms were ranked by significance based on an enrichment threshold of pFDR < 0.01 with a minimum of 3 significant genes per GO term (Supplementary Tables S14 to S18). For 3R4F, 314 GO terms were enriched for up-regulated genes with the top terms relating to “inflammatory response” (pFDR = 5.23e^−11^), “response to wounding” (pFDR = 4.05e^−10^), “migration” (pFDR = 1.42e^−09^) and “response to chemical/xenobiotics” (pFDR = 1.65e^−09^) (Supplementary Table S14). 238 GO terms were enriched for down-regulated genes with the top terms relating to “cell adhesion” (pFDR = 1.37e^−13^), “cell-cell signalling” (pFDR = 2.02e^−08^) and “development” (pFDR = 6.31e^−13^) (Supplementary Table S15). For THS, the up-regulated genes contributed to 223 enriched GO biological process terms mostly related to “immune response” (pFDR = 3.32e^−05^), “response to stress” (pFDR = 4.45e^−05^), “angiogenesis” (VEGF pathway) (pFDR = 4.47e^−05^) and “cell cycle” (pFDR = 6.93e^−05^) (Supplementary Table S16). Only 2 GO terms were significantly enriched with the down-regulated genes (Supplementary Table S17). THP 1.0 up-regulated genes were enriched for 139 terms relating to “response to organic cyclic and nitrogen compounds/xenobiotics” (pFDR = 2.10e^−04^, 2.26e^−04^), “tissue development” (pFDR = 6.70e^−05^) and “cell differentiation” (pFDR = 2.10e^−04^) (Supplementary Table S18). The down-regulated genes had no GO terms associations. A further targeted enrichment analysis was performed with the same sets of RNA features using 5 ontologies that have been associated to COPD^26^, namely: “cell adhesion” (GO:0007155), “angiogenesis” (GO:0001525), “response to organic substance/xenobiotics” (GO:0010035), “inflammatory response” (GO:0006954), “response to wounding” (GO:0009611). GO cluster plots were generated to visualize the enrichment output (Fig. 6). The GO cluster plots showed a major contribution to “cell adhesion” for the down regulated genes after 3R4F smoke exposure (74 genes), while the up-regulated genes were mostly split between “inflammatory response” (33 genes) and “response to wounding” (42 genes) (pFDR < 0.05, [FC] > 2 adj time) (Fig. 6A). For the two THPs, most genes belonging to the selected ontologies were up-regulated and participated to “response to wounding”, the THS having a total of 11 genes (at pFDR < 0.05; [FC] > 1.5 adj time) and THP1.0 a total of 7 genes (at pFDR < 0.3, adj for time) upregulated in this category (Fig. 6B and C).

### Transcriptome correlation and qPCR validation in multiple donors

**Figure 7:**
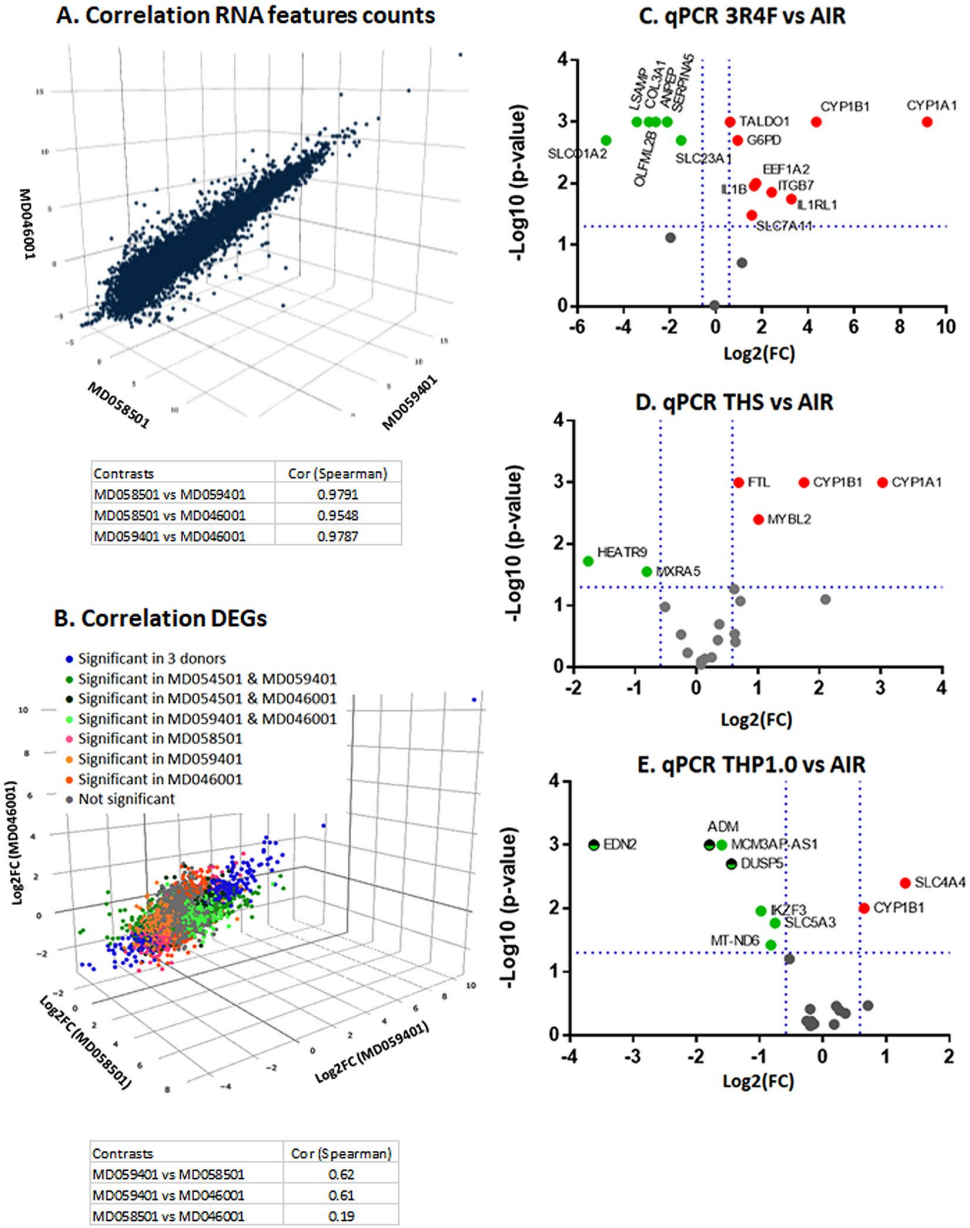
Gene expression validation in multiple donors. (**A**). Correlation plot of the normalized RNA-counts in the air controls from MucilAir™ donor MD059401 used in this study and MucilAir™ donor MD058501 used in a previous study^13^ and donor MD046001 from an unpublished study (raw sequence files provided). (**B**). Correlation plot of the Log2(FC) of the expressed genes found in common in the 3 MucilAir™ at 24 hrs post-exposure to cigarette smoke. Cells from MD58501 and MD059401 were exposed to smoke from 3R4F for an hour, cells from MD046001 were exposed to 1R6F cigarette smoke for an hour. The blue label indicates DEG (differentially expressed genes pFDR < 0.05) significant in the 3 donors, green labels have been used for DEGs significant in two donors, orange/pink labels have been used for DEGs significant in one donor, and the grey label have been used for gene not differentially expressed. Volcano plots for the qPCR validations of sets of 20 genes selected from the RNA-seq data performed with three donors showing 3R4F vs air (**C**) THS vs air (**D**) and THP1.0 vs air (**E**). The doted blue lines show the −log10(p-value = 0.05) and log2(FC = ±1.5) thresholds. Genes that were significant at these thresholds are annotated on the plots and colored in red for up-regulation and green for down-regulation.

Since one MucilAir™ donor was used for the RNA-seq experiment, we verified that this donor transcriptome was consistent with others. The correlation of the normalized RNA counts for each RNA feature quantified in the air control from this study (donor MD059401) were assessed against two donors (nasal cells) used in other studies with cigarette smoke exposure (donor MD046001 and donor MD058501). One study using donor MD058501 is published^13^ with the raw sequence file accessible at: https://www.ncbi.nlm.nih.gov/sra/SRP096285. The second study using donor MD046001 is unpublished but followed the same exposure protocol using a 1R6F^27^ reference cigarette which has similar tar delivery to 3R4F, with the raw sequence files accessible at: https://www.ncbi.nlm.nih.gov/sra/SRP126705. The RNA counts in the air controls were highly correlated between these donors with pairwise Pearson correlation coefficients between 0.98 and 0.95 (Fig. 7A). In addition, pairwise Pearson correlation between the three donors (MD059401, MD046001, and MD058501) were conducted comparing the Log2(fold change) of the RNA features at 24 hrs post-exposure to cigarette smoke. The transcriptional response from the donor used in this study (MD059401) was well correlated with donor MD046001 (Spearman corr coef = 0.60) and donor MD058501 (Spearman corr coef = 0.62) from the two other studies (Fig. 7B). It was noted however that the correlation between donors MD4046001 and MD058501 was less significant (Spearman corr coef = 0.19) possibly due to interindividual variation. The full list of RNA features expressed in the 3 donors is given in Supplementary Table S19 including fold changes and pFDR. Venn diagrams are also provided in Supplementary Figure S4 showing common differential RNA features at pFDR < 0.05 in the 3 donors.

To further verify the RNA-seq results, a qPCR experiment was conducted using 3 donors (MD048401, MD060201, MD064701) assessing 20 genes selected from the RNA-seq differential expression data, for each product treatment. The genes were selected from down and up-regulated RNA features at pFDR < 0.05, [FC] > 2 for 3R4F, pFDR < 0.05, [FC] > 1.5 for THS, and pFDR < 0.3 for THP1.0 from the time adjusted data. Three inserts per treatment and per donor were used and one exposure run was performed according to the experimental design presented in Fig. 1D (Figure adapted from Haswell *et al*.^13^) and RNA samples were collected at 24 hrs post-exposure. The nicotine measured in the media from the exposure chamber was consistent with our RNA-seq experiment with higher nicotine delivered from the THPs (Air: 22.9 ng/ml; 3R4F: 5780 ng/ml; THS: 8390 ng/ml; THP1.0: 6380 ng/ml). The reduction in nicotine delivery from the THS compared to our first exposure runs could be due to the use of a different batch of device. The volcano plots in Fig. 7C to E show the genes that were differentially expressed at a p < 0.05, [FC] > 1.5 thresholds for each treatment. With 3R4F smoke exposure, 16 genes were confirmed to be differentially expressed out of 20 (Fig. 7C). All 16 were matched for up and downregulation with the RNA-seq data. No amplification was detected for one mRNA, PAPLN. Three other mRNA CRCT1, PCDH18, and CXCL13 did not reach significance due to donor variability. Both THPs only had 6 genes matching the RNA-seq data (Fig. 7D and E), and 3 significant RNAs, being ADM, DUSP5, EDN2 for the THP1.0 were not in agreement with the RNA-seq fold change direction (Fig. 7E). CYP1A1 and CYP1B1 were increased by 8- and 3-fold respectively, following THS aerosol exposure and CYP1B1 1.6-fold for the THP1.0. Details of the qPCR values are given in Supplementary Table S20.

### Inflammatory markers in multiple donors

**Figure 8:**
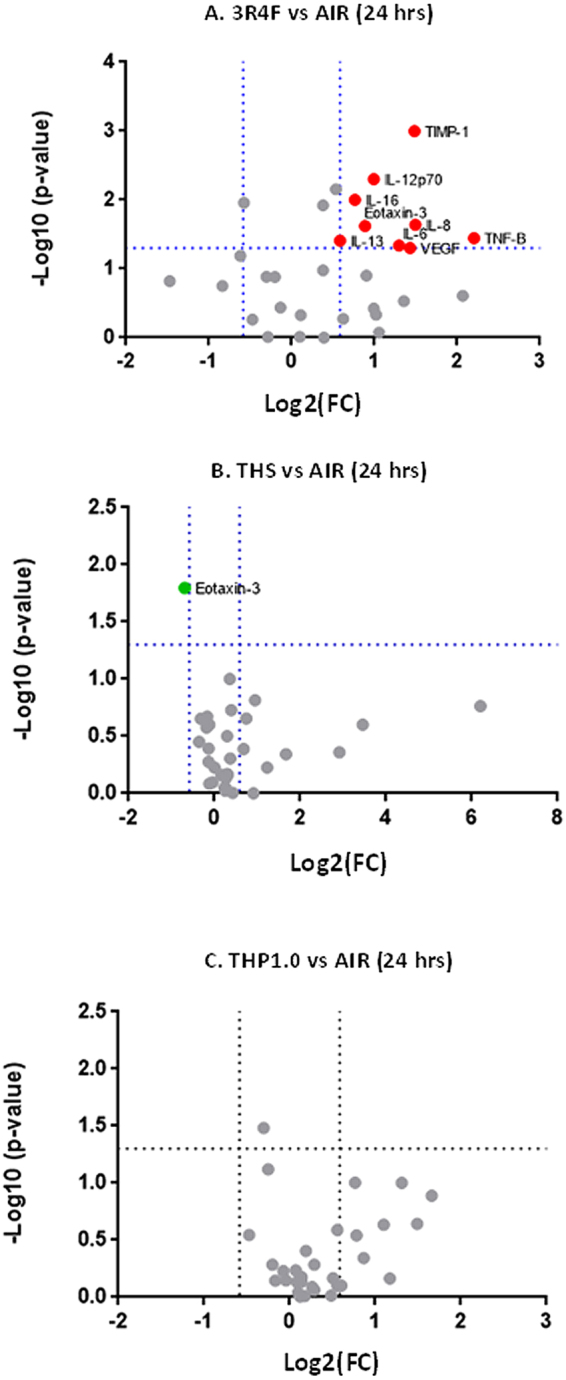
Volcano plots for a panel of 34 cytokines and MMPs quantified in the supernatants from three donors comparing the following treatments: 3R4F vs air (**A**) THS vs air (**B**) and THP1.0 vs air (**C**). The doted blue lines show the −log10(p-value = 0.05) and log2(FC = ±1.5) thresholds. Markers that were significant at these thresholds are annotated on the plots and colored in red for increase and green for decrease.

“response to wounding” was an enrichment term in common to all the tested aerosols and “inflammatory response” was significant for the 3R4F and THS. To verify the potential impact of the different aerosol exposure on inflammation markers, we collected the cell media from the qPCR exposure run (24 hrs post-exposure, 3 donors) and quantified a panel of 31 cytokines and 3 tissue remodeling MMPs. The volcano plots in Fig. 8 present the cytokines that were significantly (p < 0.05) increased or decreased [FC] > 1.5 in the culture media 24 hrs after exposure to cigarette smoke (Fig. 8A) and the THPs (THS and THP1.0) aerosols (Fig. 8B and C). Nine cytokines were changed after cigarette smoke exposure, including IL-13, IL-6 and IL-8. Eotaxin-3 was significantly decreased after exposure to the THS aerosol only. No significant changes were observed for the THP1.0 at 24 hrs post exposure.

## Discussion

RNA-seq-based transcriptomics has previously been used to evaluate the impact of aerosols generated by different electronic cigarettes on 3D lung tissue models, showing a reduced, yet not neutral, effect on the cells compared to cigarette smoke^13,14^. Similarly, the multiplicity of THP designs based on heating temperature, consumable format and blend, can result in different and simpler aerosol chemistries^5,17^ compared to conventional tobacco products. Systems toxicology assessment of the biological impact of a THP aerosol has been previously reported to demonstrate reduced transcriptomics, proteomics, and functional marker perturbations in *in vitro* and animal models^15,16^. Thus, omics based assessments have shown potential to detect discreet biological changes that can be missed by more targeted assays. No studies have currently evaluated multiple THP devices since THPs represent a category of products. In this study, we used a systems biology approach to examine the global transcriptional response of a 3D airway cell model (MucilAir™) following exposure to two commercially available THPs: THS consisting of a king size tobacco consumable heated with a central blade up to 350 °C^3^, and THP1.0 comprising a slim size consumable heated peripherally up to 240 ^○^C^17^. For comparison, a 3R4F cigarette and an air control were included in the study. In these experiments nicotine which is an aerosol ingredient in common between all products was used as dose surrogate to select our dilutions. Using a Borgwaldt RM20S smoking engine, a 1/30 smoke dilution was selected for the 3R4F exposure; we have previously reported this dilution to correspond with that of typical human exposures^18^. For the THPs the aerosol dilution and puff numbers were matched to compare products on a consumable basis. The single 1/5 dilution used for THPs aimed at delivering higher nicotine compared to 3R4F, to represent an extreme scenario of nicotine titration by consumers when they switch products^28,29^. An intense smoking regimen (HCI) was applied because other standard smoking regimens such as ISO, tend to underestimate the in-use yields of toxicants^30^. The MucilAir™ cells from one donor were exposed to the aerosols at the ALI then given 24 and 48 hrs recovery after which multiple endpoints were assessed including gene expression.

The nicotine concentration measured immediately after exposure indicated that the THS at 1/5 dilution (14866.7 ± 709.5 ng/ml) was approximately double that of 3R4F at 1/30 dilution (6528 ± 431.6). The THP1.0 delivered marginally (7912.5 ± 763.6), but significantly (p = 0.034) higher nicotine than 3R4F (Fig. 2). Forster *et al* (2017) reported mainstream aerosol yields of nicotine of 0.462 ± 0.037 mg/consumable^17^ for the THP1.0 and 1.32 ± 0.16 mg/consumable^5^ for the THS compared to 3R4F (2.02 ± 0.08 mg/consumable^17^) smoked at HCI. Those values were in good agreement with the nicotine ratios observed in our exposure chambers at the smoke dilution used. The nicotine yields measured for the two THPs are potentially associated with their different operating temperature, format and blend of their tobacco consumables.

We chose to use MucilAir™ of nasal origin because the tissue is easier to source and there is a wealth of evidence showing that nasal cells are good surrogates for bronchial cells *in vitro* and in clinical samples^31–34^. Notably the Spira group demonstrated that the differential gene expression in tissue from nasal origin and bronchial origin were correlated^33^. Furthermore, it has also been shown that smoking creates a field of molecular injuries throughout the airways and a good concordance was found between cancer- and COPD-associated genes in bronchial/large airways and nasal tissues from diseased subjects^31,35^. The percentage of each cell subpopulation we report here is in line with what has been reported in the literature for bronchial and nasal epithelium with approximately 60–70% of FOXJ1 positive ciliated cells, 20–25% of p63 positive basal cells, and 2–3% MUC5AC positive goblet cells in the air control^13,15^ (Fig. 3). Functional cilia were also detected using the SAVA system (Fig. 3). MucilAir™ morphological markers assessed at 24 hrs and 48 hrs post-exposure were not affected by the two THPs except for MUC5AC at 24hrs after exposure to the THP1.0 aerosol (Fig. 3B to D). The observed drop (p < 0.01) in the MUC5AC cell population from 2.7 ± 1.3% in the air control to 0.8 ± 0.5% in the THP1.0 aerosol treated cells was not observed at 48 hrs (Fig. 3B). Typically, MUC5AC positive cells only represent a small fraction of the upper respiratory epithelium, therefore small changes that could happen by chance can have a disproportionate effect on this marker and thus caution is advised.

3R4F smoke exposure affected a series of functional endpoints at 48 hrs with significant reduction (p < 0.05) in CBF (7.7 ± 0.43 hz in air *vs* 6.2 ± 1.0 hz with 3R4F) (Fig. 3A), percentage of active ciliated area (65.1 ± 12.6 % in air *vs* 36.5 ± 29.5 % with 3R4F) (Supplementary Figure S1), TEER (1814.7 ± 202.9 Ω in air *vs* 1384.2 ± 413.4 Ω with 3R4F)(Supplementary Figure S2), and a significant (p < 0.05) LDH release (4.23 ± 2.95 % with 3R4F *vs* 1.22 ± 0.99 % in air) (Supplementary Figure S3). Overall, there were no changes observed for these endpoints when the air control was compared to either THPs aerosol treatments (Fig. 3A and Supplementary Figure S1 to S3), however, the CBF increased at 24 hrs post-treatment only with the THS aerosol (Fig. 3A). A slight increase in CBF with exposure to the THS has previously been reported as a function of the aerosol concentration shortly after exposure (4 hrs) and a slight decrease after 24 hrs^36^. Thus, our data together with the historical data does not point towards a clear upward or downward trend for CBF after THS aerosol exposure.

Thousands of RNA features were differentially expressed after 3R4F cigarette smoke exposure both at the 24 hrs (2806 RNAs) and 48 hrs (2727 RNAs) time points (pFDR < 0.05, [FC] > 1.5) (Fig. 4, Supplementary Table S1). Fewer RNA features were altered by the THP aerosols exposure using the same pFDR and FC thresholds. Interestingly, a distinct differential RNA response was observed between the THS and the THP1.0 with 65, 66, and 115 RNAs significant for the THS at 24, 48 hrs, and adjusted for time, respectively, and only 2 significant RNAs for the THP1.0 post-exposure with time adjustment. The number of RNA features increased to 71 for the THP1.0 but only by applying a no fold change threshold and a pFDR cut off of 0.3 in the post-exposure time adjusted data (combining the data from the two time points to increase statistical power) (Fig. 4, Supplementary Table S1). We cannot conclude that the different transcriptional response observed between THPs is due to the different nicotine delivery. We used nicotine as a dose surrogate knowing that several tobacco smoke HPHCs (Harmful and Potentially Harmful Constituents) can be generated at low temperature and are correlated with nicotine. For instance, some aldehydes have been shown to follow a linear and other a non-linear correlation with nicotine in the THS aerosol at ISO smoking regime^37^. Those HPHCs can be responsible for the observed response and can be detected at low levels in the aerosol of the THPs tested in this study. For example, 2.2 μg and 11.3 μg of Acrolein can be detected at HCI smoking regime per THP1.0 and THS tobacco consumable, respectively. Furthermore, both CYP1A1 and CYP1B1 are induced by the THS aerosol and aerosol chemicals such as PAHs (polyaromatic hydrocarbons) are known potent inducers of CYP1A1 and CYP1B1^38^ whilst CYP1A1 induction by nicotine has only been anecdotally reported in rats and not reported in humans^39^.

Since this is the first transcriptomics study on the THP1.0, there is currently no available data to compare our results with existing historical data, however microarray data has been published for the THS. Iskandar *et al* reported 91 differentially expressed genes in MucilAir™ 24 hrs after exposure to the THS aerosol with dose equivalent of nicotine doubled compared to 3R4F at pFDR < 0.05 with no fold change threshold^15^. In our study, 13 RNA features overlapped between the THS (115 RNAs, thresholds pFDR < 0.05, FC > [1.5], adjusted for time) and THP1.0. (71 RNAs, thresholds pFDR < 0.3, adjusted for time) of which two were CYPs genes (CYP1B1, CYP26A1).

Enrichment analyses revealed a series of perturbations across a variety of biological processes different in nature and in amplitude based on the product tested:

(i) For 3R4F smoke, the enrichment was conducted with RNA features selected at pFDR < 0.05 and [FC] > 2 (adjusted for time) (Supplementary Table S5). “Inflammation” (pFDR = 5.23e^−11^), “response to wounding” (pFDR = 4.05e^−10^), “response to chemicals/xenobiotics” (pFDR = 1.65e^−09^), and “cell migration” (pFDR = 1.42e^−09^) were highly significant (Supplementary Table S14). Such response is consistent with previous *in vitro* studies^13,15^ and the airway physiopathology of cigarette smoking^40^. This was further illustrated when we conducted an enrichment analysis focused on 5 ontologies that have been associated with COPD across multiple studies^26^. Our resulting cluster plots and also the SRA genesets^23^ analyses (Figs 5 and 6A) highlighted a clear downregulation of genes contributing to cell adhesion. In addition, a series of inflammatory response genes were upregulated, with notably, the genes coding for the pro-inflammatory interleukin 1A, 1B and 6, together with the IL1RN agonist and IL-1 receptor IL1RL1. When three subjects were compared, the IL-6, IL-1α, IL-13 and IL-8 proteins were also found increased by more than 2-fold in the culture media at 24 hrs post-exposure, of which IL-13, IL-6 and IL-8 were statistically significant (Fig. 8). IL-8 regulates the production of elastase and plays a key role in conditions such as fibrosis^41^ whilst IL-13 is a key driver of goblet cells hyperplasia. The combined changes in the IL-1 pathway with IL-13, IL-6, and IL-8 protein secretion is consistent with what has been reported previously *in vitro* and in COPD^15^. COPD, however, is a complex long term multi-stage disease and therefore cannot be replicated as such *in vitro* using an acute exposure.

(ii) For the THS, enrichment was performed with the RNA features significant at pFDR < 0.05 and [FC] > 1.5 (adjusted for time) (Supplementary Table S8). Focused analyses on GO terms related to COPD highlighted an over-representation of “response to wounding” (pFDR = 8.18e^−04^) based on 9 up-regulated RNA features (Fig. 6B and Supplementary Table S8). Treatment-based clustering is also observed for the THS in the SRA heatmap for “wound healing” driven by the upregulation of TIMP1, IL1B, IL6 and downregulation of COL3A1 and COL1A2 (Fig. 5B,C). The COPD-related GO term “inflammatory response” was also significant for the THS at a pFDR = 2.93e^−3^ (Fig. 6B and Supplementary Table S16). IL6, IL1A, and IL1B genes were increased by values close to, or higher than 2-folds after exposure to the THS aerosol, however, no increase in the corresponding secreted cytokines was observed when assessed in three donors at 24 hrs (Fig. 8B). The other top GO terms in the THS-treatment group with up-regulated genes included “immune response” (pFDR = 3.32e^−05^), “cell cycle” (pFDR = 6.93e^−05^) “response to stress” (pFDR = 4.45e^−05^), and “angiogenesis” (pFDR = 4.47e^−05^) (Supplementary Table S16). There was a great degree of overlapping genes across those terms including IL1A, IL1B, IL6, GPX2, CYP1B1, CYP1A1, CYP26A1, TIMP1, IGFBP6, FOSL1. CYP1A1 and CYP1B1 are also two main drivers of the aerosol treatment-based clustering observed in the SRA Phase I xenobiotics metabolism heatmap (Fig. 5C) and were confirmed in the independent qPCR repeat, whilst CYP26A1 was borderline significant when tested by qPCR (Fig. 7B). In two previous studies, Iskandar *et al* reported data consistent with our analysis with statistically significant impact of the THS aerosol on xenobiotic metabolism pathways, inflammatory processes, and cell cycle also with a nicotine dose double that of 3R4F^15,36^. The amplitude of the gene network perturbations by the THS aerosol was strongest at 4 hrs post-exposure but still significant at either 24 or 48 hrs post-exposure. It was, however, markedly reduced compared to 3R4F smoke.

(iii) For the THP1.0, only two genes (EDN2, CYP26A1) were differentially expressed at pFDR < 0.05 and [FC] > 1.5 (adjusted for time) (Supplementary Table S11), hence no GO terms enrichment could be derived. The analysis ran with the 71 RNA features identified at the looser threshold of pFDR < 0.3 and no fold change filter (Supplementary Table S12), identifying enrichments in GO terms only for the up-regulated genes including “tissue development” (pFDR = 6.7e^−05^), “cell differentiation” (pFDR = 2.1e^−04^), “xenobiotic and nitrogen metabolism” (pFDR = 2.10e^−04^ to 2.26e^−04^) (Supplementary Table S18). Similar to the THS, “response to wounding” was also identified in the COPD-focused analysis based on 7 genes. The significance threshold for enrichment, however had to be raised to pFDR < 0.05 (pFDR = 1.37e^−2^) instead of 0.01 (Fig. 6C, Supplementary Table S18). The COPD-related GO term “inflammatory response” was not significant for THP1.0. genes and no cytokines were found to be changed in the culture media at 24 hrs. A series of genes overlapped between these different pathways with notably ADM, CTGF, CYP1B1, CYP26A1, HRAS, JUN, JUNB, and JUND and of those CYP1B1, CYP26A1, and JUN were also in common with the THS. JUN and FOS differential RNA expression has also been reported with exposure to the THS aerosol but mostly at 4 hrs post-exposure^15,36^. JUN and FOS are the heteromeric elements of the transcription factor AP-1 which is activated by reactive oxygen species and particulate matter. This dimeric transcription factor regulates the expression of antioxidant enzymes, extracellular matrix proteins, and epithelial secretory enzymes^42^.

These enrichment results based on the RNA-seq data should be put in perspective for the THP1.0 aerosol as none of those genes had a fold change greater than 1.5. For the THS many genes were close to the 1.5-fold change threshold, with CYP1A1 and CYP1B1 being the strongest responders with 10 and 3 times fold change, respectively. In comparison, 3R4F smoke caused a 28, and 1087-fold induction of CYP1B1, and CYP1A1 mRNAs in the time adjusted contrast, respectively.

Few of the genes that were included in the multiple donor qPCR experiments could confirm the THPs RNA-seq results (Fig. 7D and E). After THP aerosols’ exposure using a single donor many of the RNA-seq expression fold changes were between 1.5 and 2.0. It is likely that the increased variability from multiple donors has a greater impact on the statistical significance of genes with a small fold change. It is therefore advisable to increase the statistical power by using more donors or replicates when assessing the cellular response from two simple aerosols. Nevertheless, the qPCR performed with 3 donors and RNA-seq data concur to a reduced transcriptional impact of the THS compared to 3R4F (Fig. 7C and D). It is worth noting that the qPCR results also confirmed the greater induction of CYP1A1 and CYP1B1 with the THS and the more limited increase of CYP1B1 expression with the THP1.0 aerosol. Induction of CYP genes such as CYP1A1 and CYP1B1 is typically regulated by the AHR/ARNT transcription factor. AHR/ARNT activation in the context of inhaled tobacco smoke is often an indication of the presence of PAHs^43^, therefore the amplitude of CYP1A1, CYP1B1 induction potentially points towards product-specific aerosol chemistries.

Since we used one donor for the RNA-seq work, we have not assessed how variable is the transcriptomic response in MucilAir™ from different subjects and caution is warranted, in particular, when considering the limited response from THP exposure. Thus, the RNA feature counts in the air controls and the differential expression in response to cigarette smoke measured in this study were compared to two other MucilAir™ donors for which we had historical data after performing smoke exposures of identical intensities (Fig. 7A and B). The transcriptome of the donor used for the RNA-seq in this study appeared consistent with two other donors (Fig. 7A) since a comparison of the air control normalized RNA-counts gave robust Pearson correlation coefficients 0.95–0.98. Furthermore, a Pearson coefficient of 0.6 was also obtained when the correlation was performed to compare the fold changes of RNA-features in common in the donor used in this study and two donors treated with cigarette smoke from historical studies (Fig. 7B). In addition, the 3R4F exposure qPCR results repeated with 3 different donors were in very good agreement with the RNA-seq data with 16 genes matching out of 20 selected candidates (Fig. 7C). These tests suggest that the MucilAir™ used in the RNA-seq part of this study were sourced from a subject that responded consistently to what has been observed with two other MucilAir™ donors from previous studies^13^. Greater interindividual variability was observed when a pairwise comparison was performed only between the two donors for which we had historical data^13^ (Peasron correlation of 0.19) (Fig. 7B).

One limitation of this work was first the use of an acute exposure which does not reflect long term exposure to the THP aerosols, and the absence of a short post-exposure time point. On this note, acute studies performed at 1 hr post exposure with e-cigarettes and at 4 hrs post-exposure with the THS have reported moderate transcriptional responses^15,36^. In the case of e-cigarette aerosol exposure, Shen *et al* reported more downregulated genes with e-liquid containing nicotine compared to e-liquid without nicotine at 1 hour post exposure^14^. The reported early responses did not appear sustained since they reported no differences at 24 hrs. It is unclear whether this observation translates into functional changes, however, this shows that there is value in adding early time points. Second, MucilAir™ of nasal origin have been shown to give similar response to the large airway epithelium from COPD patients, yet it differs from the response observed in the small airways^35^. Although MucilAir™ are metabolically active, the small airway contains a larger number of Club cells^44^ responsible for detoxification and potentially bioacativation of chemicals which could drive a different response. Therefore, it might be of value to perform similar assessment in small airways models such as SmallAir™^44^. Finally, we only measured cytokines at 24 hrs post exposure in the basal media, but it would also be interesting to assess the cytokine secretion on the apical compartment of the MucilAir™ at multiple time points, including immediately after the exposure.

In conclusion, RNA-seq based transcriptomics showed a reduced impact of THP aerosols on differential expression in MucilAir^TM^ compared to cigarette smoke. Enrichment analyses and cytokine profiling confirmed a strong inflammatory, cell adhesion and response to wounding component in the 3R4F cigarette smoke treatment. Response to wounding and xenobiotics metabolism were also found to be common enriched ontologies for the THPs but this was not accompanied by inflammatory cytokine secretion at the tested time point. The acute exposure used here represents an extreme event; however, the limited transcriptional response at 24hrs and 48hrs post-exposure to THPs’ aerosols warrants future studies. Repeated exposure over a period of days or weeks might better represent the normal exposure scenario and is potentially possible in cells such as the MucilAir™ which can be kept in culture for extended periods of time^45^.

## Materials and Methods

### Cell culture

Reconstituted 3D human airway epithelia MucilAir™ cells (Epithelix Sarl, Genève, Switzerland) were used in this study. Cells were isolated by the manufacturer from nasal swabs after obtaining permission by informed consent in compliance with the Declaration of Helsinki on biomedical research including anonymity of the samples, and approval from local ethics commission and the Geneva Canton Department of Economy and Health to be used for research purpose. Once collected, cells were passaged before seeding on a Transwell™ culture insert and differentiated at the air-liquid interface. Upon delivery, cells were acclimatized for a week and the proprietary media was changed every 2 to 3 days. MucilAir™ cells were maintained in a humidified incubator at 37 °C with 5% CO_2_. Cells from donor MD059401 were used for the RNA sequencing workflow (Fig. 1C) (Figure adapted from Haswell *et al*.^13^) and cells from 3 donors MD048401, MD060201, MD064701, were used for the qPCR workflow (Fig. 1D) (Figure adapted from Haswell *et al*.^13^). MucilAir™ are passaged cells *in vitro* and therefore are not deemed relevant material under the UK Human Tissue Act 2004 which regulates storage, use and disposal of human tissue^46^.

### Experimental design

Two series of experiments were performed. First a single donor (MD059401) was used for the RNA-seq workstream (Fig. 1C) (Figure adapted from Haswell *et al*.^13^), then three donors (MD048401, MD060201, MD064701) were subsequently used for the qPCR validation and cytokine workstream (Fig. 1D) (Figure adapted from Haswell *et al*.^13^). For both series, the cells were exposed at the air liquid interface for one hour (Fig. 1B) (Figure adapted from Haswell *et al*.^13^) either to aerosols generated from the tobacco products aerosols or to filtered air following the exposure protocol described below.

For the RNA-seq, each treatment and time point had 3 technical replicates for the RNA extractions, and 3 for the immunohistochemistry. Each experiment was repeated a minimum of 3 times independently to generate a total of 9 samples per treatment and per time point (3 technical replicates in 3 independent repeats of the exposure runs). A fourth exposure run was performed to replace damaged samples during manipulation from the previous runs (eg TEER measurement washes). Each aerosol exposure run was performed for one hour and run numbers were recorded as covariates. Immediately after each exposure run the media from the exposure chambers were collected for nicotine quantification. The cell inserts were placed in fresh media and returned to the incubators for 24 and 48 hrs. At 24 and 48 hrs post-exposure, non-destructive endpoints including CBF, TEER, and cytotoxicity were quantified. Half the inserts were then fixed for immunohistochemistry and the other half lysed for RNA extraction.

For the qPCR validation, three different MucilAir™ donors were used in a single exposure run with three technical replicates and one 24 hrs post-exposure time point (total of 9 samples per treatment) (Fig. 1D) (Figure adapted from Haswell *et al*.^13^). The same endpoints and samples were collected following the same steps as previously described. In addition, the cell media was stored for cytokine profiling.

### Products

Three products were used in this study, a reference 3R4F combustible cigarette (University of Kentucky) and two commercially available THPs (THP1.0 and THS) (Fig. 1A). The 3R4F is a 9.5 mg ISO tar yield cigarette. The THP1.0 device use a slim size tobacco rod inserted in a heated casing delivering heat below 250 °C from the periphery^4^. The casing also includes an Li-ion battery and heat sensors and built-in controls. The THS use a king size tobacco consumable inserted on a heated blade not exceeding 350 ^○^C located in the center of the holder^3,5^.

### Smoking regimens and exposure protocol

All product aerosols were generated on a Borgwaldt RM20S smoking engine (Hamburg, Germany, serial number: 0508432) and delivered to MucilAir^TM^ inserts at the air-liquid interface in modified exposure chambers (Fig. 1B) (Figure adapted from Haswell *et al*.^13^). The 3R4F cigarette were smoked at the HCI standard regimen^47^ (55 ml puff over 2 seconds, every 30 seconds, with filter vents blocked and a bell-wave puff profile) for one hour at a 1/30 dilution: The 1/30 smoke dilution was used based on the estimated smoke dilution in the human airways^13,18^. The THPs were tested using a modified HCI (mHCI) smoking regimen (55 ml puff volume, 3 s puff duration, 30 s puff intervals and a bell-wave puff profile) at a 1/5 dilution with 30 sec pre-heating for the THS and 40 seconds pre-heating for the THP1.0T. Exposures were conducted for 1 hr and puff numbers were matched between the different products. A common THPs aerosol dilution of 1/5 was selected to exceed the nicotine of 3R4F cigarette based on nicotine quantified in the media from the exposure chamber. Air controls were performed with cells exposed to an intermittent flow of sterile air in an exposure chamber, at a frequency and duration identical as treated cells.

### Nicotine quantification

The nicotine quantification method has been described in Haswell *et al*.^13^. Briefly nicotine in the cell culture media from the exposure chamber is quantified using a Waters Acquity UPLC (Milford, USA) connected to a AB SCIEX/API 4000 Q-Trap® mass spectrometer (Framingham, USA). The media is spiked with a d4-nicotine internal standard (Sigma Aldrich, St Louis, USA) at 10 ng/ml before being vacuumed dried and reconstituted in 1 ml 5% acetonitrile:water solution (v/v). The UPLC and MS methods were adapted from Onoue *et al*., and Jin *et al*., respectively^48,49^.

### CBF

CBF was quantified using the Sisson Ammons Video Analysis (SAVA) system (Ammons Engineering, Clio, USA)^19^, using an inverted AX 10 Zeiss microscope (Gottingen, Germany) fitted with a heated XLmulti S1 chamber and a Basler scA640-120fm digital camera (Ahrensburg, Germany) as previously described (19). The microscope chamber temperature is maintained at 37 °C during the data acquisition process. CBF was measured at either 24 or 48 hrs post-exposure, two readings per MucilAir™ insert were taken at 120 frames per sec with a total of 512 frames per reading. The SAVA software was then used to calculate the percentage active area and CBF for each sample.

### TEER

200 μl pre-warmed PBS was added to the apical surface of the MucilAir™ and the TEER was measured using World Precision Instruments STX2 electrodes and EVOM^2^ epithelial voltometer (Sarasota, USA).

### Immunohistochemistry

Immunohistochemistry was conducted on MucilAir™ inserts fixed with 3.6% formaldehyde/PBS, followed by a dehydration step using 70% ethanol and paraffin inclusion. Tissue sections (4 µm) were incubated with the primary monoclonal antibodies (MUC5AC clone 45M1, FOXJ1 clone 3–19, p63 clone 4A4, Abcam, Cambridge, USA) for 1 hr at room temperature. After 3 washes the biotinylated secondary antibody (Dako, Ely, UK) was used to detect the primary, and visualized using an HRP-conjugated ABC system. Quantitative image analysis was performed using ImagePro Plus6.2 (Media Cybernetics, Cambridge, UK). Twenty images spanning the entire length of the section were acquired per insert to obtain a composite % for MUC5AC, FOXJ1 and p63.

### Cytotoxicity

The Promega CytoTox-ONE™ Homogeneous Membrane Integrity Assay (Madison, USA) was carried out to assess cytotoxicity by measuring lactate dehydrogenase (LDH) release in the cell culture media. Assays were performed in accordance with the manufacturer’s instructions and results were read using a Molecular Devices SpectraMax M3 plate reader (Sunnyvale, USA). An arbitrary acceptance criteria of 5% average cytotoxicity per treatment and no more than 10% cytotoxicity per insert was defined.

### RNA isolation

The RNA was isolated using the QIAGEN miRNeasy Mini kit (Hilden, Germany) according to the manufacturer’s instructions. Cells were directly lysed on the insert using QIAzol lysis reagent. The RNA extraction protocol was performed on the randomized lysates by one operator either manually for the RNA-seq samples or using the QIAcube robotic workstation for the qPCR validation samples. The RNA samples were analyzed using an Agilent Bioanalyzer 2100 (Santa Clara, USA) with a RIN acceptance criteria equal or greater than 8.0 and quantified using a NanoDrop ND1000 spectrophotometer (Wilmington, USA).

### RNA-seq and qPCR

The RNA sequencing was conducted as described in Haswell *et al*.^13^. Briefly, the RNA libraries were prepared using the Ribo-Zero Illumina® TruSeq Stranded Total RNA kit (Illumina®, San Diego, CA, USA). The libraries were QC checked for cDNA size and distribution using a Bioanalyzer 2100 (Agilent, Wokingham, UK). Multiplexed 150 bp paired-end RNA-sequencing was performed on an Illumina® NextSeq 500 platform (Illumina®, San Diego, CA, USA) with 8 randomized samples per flow cells with a target depth of 40 million pair reads. Raw FASTQ sequence files can be found on NCBI-SRA at: https://www.ncbi.nlm.nih.gov/sra/SRP126155.

For the qPCR validation, probe sets for 20 genes ranked by fold change intensity (up and down-regulation) and statistical significance were selected for each treatment from the time adjusted RNA-seq data. Custom TaqMan array 96-well plates were designed accordingly. The cDNA was prepared using the high-capacity RNA-to-cDNA Reverse Transcription Kit (Applied Biosystems, Foster City, USA). The qPCR were run using the TaqMan Fast Universal Master mix II with UNG on a fast PCR 7500 (Applied Biosystems).

### Secreted inflammatory proteins

The levels of secreted inflammatory cytokines and MMPs were determined in the qPCR validation cell culture media samples. The following Meso Scale Discovery (Rockville, USA) kits were used: V-PLEX Human Cytokine 30-Plex (K151A0H-2), Human MMP 3-Plex Ultra-Sensitive (K15034C-2), V-PLEX Human IL-8 (K151RAD-2), V-PLEX Human VEGF (K151-RHD-2) and V-PLEX Human MCP-1 (K151NND-2), Human TIMP-1 singleplex (K151JFC-1). All assays were performed in accordance with the manufacturer’s instructions and analyzed using a SECTOR S 600 instrument.

### Statistics

The Raw sequencing files for the donor used in this study (MD059401) are available on NCBI-SRA (https://www.ncbi.nlm.nih.gov/sra/SRP126155) and the other data is presented either in supplementary materials or is available on request.

For the nicotine, functional endpoints/immunohistochemistry and cytokines, an unpaired t-test was used to perform comparisons of treatments versus air control.

For the RNA-seq, samples were QC analyzed using the arrayQualityMetrics package in Bioconductor^50^. Outliers were identified based on 4 parameters, maplot, boxplot, heatmap and manual inspection. The RNAseq data was preprocessed and aligned to the human genome (GRCh38) using STAR aligner and the number of mapped read-pairs per gene were quantified based on the GENCODE v25 annotation. The resulting count data for the samples was normalized using TMM (Trimmed Mean of M-value) and transformed with VOOM to log2-counts per million with associated precision weights. Comparisons of the treatment *vs* air control were undertaken using linear modelling. Subsequently, empirical Bayesian analysis was applied including adjustment for multiple testing, which controls for false discovery rate (FDR)^51^. The null hypothesis was that there was no difference between the groups being compared. The Bioconductor package Limma^52^ was used and the gene lists which result were fully annotated.

For qPCR validation, the Ct values were normalized against GUSB and RPLP0 to obtain ∆Ct. Statistical analysis used a t-test to compare treatments with air controls.

For the correlation, FASTQ files for donor MD046001 can be found at https://www.ncbi.nlm.nih.gov/sra/SRP126705 and FASTQ files for donor MD058501 can be found at https://www.ncbi.nlm.nih.gov/sra/SRP096285. Three-way comparisons (Pearson correlation) between donor MD059401 (this study), MD046001, MD058501 were performed with the mean normalized RNA feature counts from the air controls at 24hrs and for the Log2(FC) cigarette smoke vs air at 24 hrs post-exposure.

### Enrichment analyses

131 genesets were obtained from the SABiociences website on July 5th 2013^23^. This list of pathway-focused arrays was used to assess the fold-changes for RNA features significant at pFDR <  0.05 and [FC] > 1.5 in one or more of the comparisons and presented as heatmaps.

Significant genes for each treatment adjusted for post-exposure time (3R4F 1/30, pFDR < 0.05, [FC] > 2; THS 1/5, pFDR < 0.05, [FC] > 1.5, THP1.0, pFDR < 0.3) were analyzed for enrichment of GO terms across the Biological Process GO ontologies^25^ using a hypergeometric test. Enrichment was assessed for up- and down-regulated genes separately and filtered for pFDR < 0.01 and at least 3 genes mapping to the GO term. For the cluster plots, five COPD-related GO terms consistent across multiple studies^26^ were selected to conduct the enrichment: “cell adhesion” (GO:0007155), “angiogenesis” (GO:0001525), “response to organic substance/xenobiotics” (GO:0010035), “inflammatory response” (GO:0006954), “response to wounding” (GO:0009611). Cluster plots were generated to for each of these data sets using the GOplot package^53^ in R, to generate multi-layered visualizations of the functional analysis data.

## Electronic supplementary material

Supplementary Figures S1 to S4

Supplementary Table S13

Supplementary Tables S1 to S12 and S14 to S20
