# Supplementary material for: In vitro RNA-seq-based toxicogenomics assessment shows reduced biological effect of tobacco heating products when compared to cigarette smoke
Source: Sci Rep. 2018 Feb 5;8:1145. doi: 10.1038/s41598-018-19627-0 (PMC5799303; doi:10.1038/s41598-018-19627-0)

# *In vitro* RNA-seq-based toxicogenomics assessment shows reduced biological effect of tobacco heating products when compared to cigarette smoke

Linsey E Haswell[1], Sarah Corke[1], Ivan Verrastro[1], Andrew Baxter[1], Anisha Banerjee[1], Jason Adamson[1], Tomasz Jaunky[1], Christopher Proctor[1], Marianna Gaça[1] & Emmanuel Minet[1]*

[1]British American Tobacco R&D Centre, Regents Park Road, Southampton, SO15 8TL UK

*Corresponding author: emmanuel_minet@BAT.com

**Supplementary Figure S1:** Scatter plot of MucilAir™ percentage of ciliated active area (AA) after exposure to air, 3R4F smoke 1/30, THS 1/5 and THP1.0 1/5 aerosol. AA at 24hrs (A) and 48hrs (B) post treatment. * denotes a significant difference versus air control at $p<0.05$.

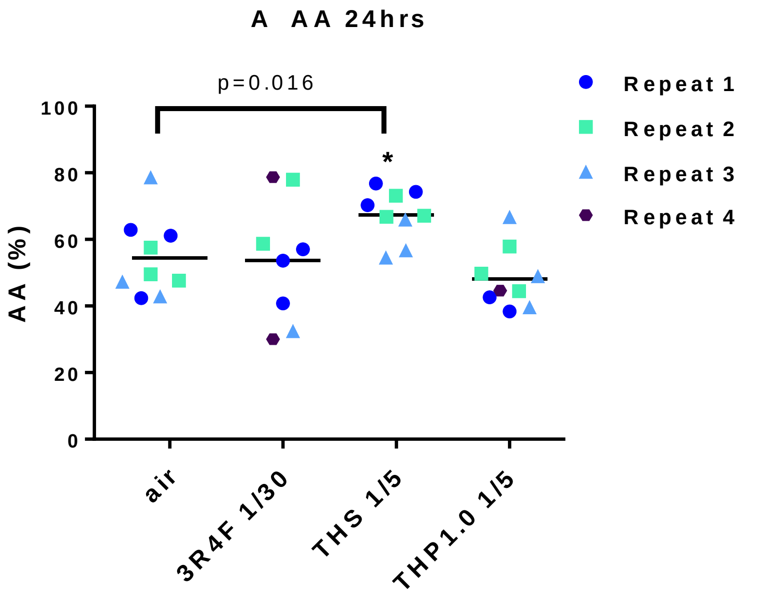


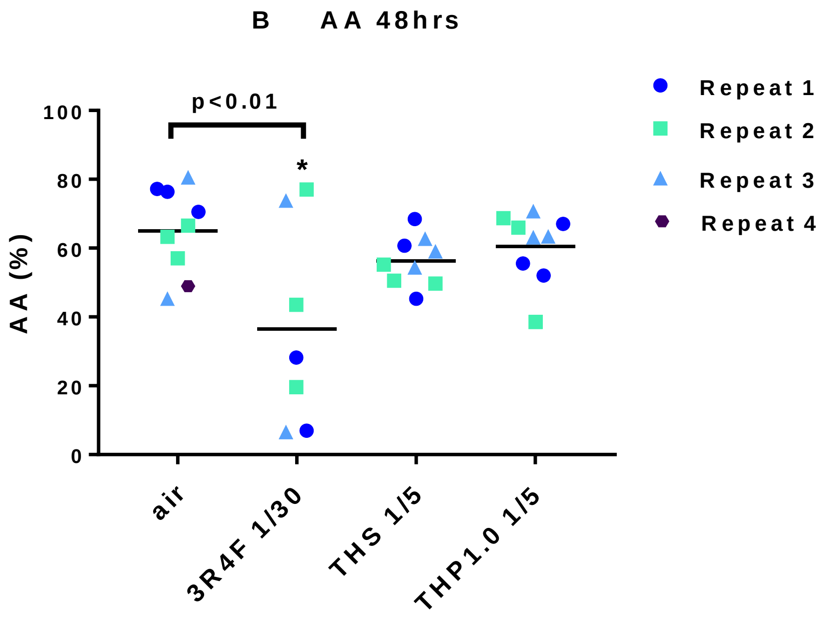

**Supplementary Figure S2:** Scatter plot of MucilAir™ trans epithelial electrical resistance (TEER) after exposure to air, 3R4F smoke 1/30, THS 1/5 and THP1.0 1/5 aerosol. TEER at 24hrs (A) and 48hrs (B) post treatment. * denotes a significant difference versus air control at p<0.05.

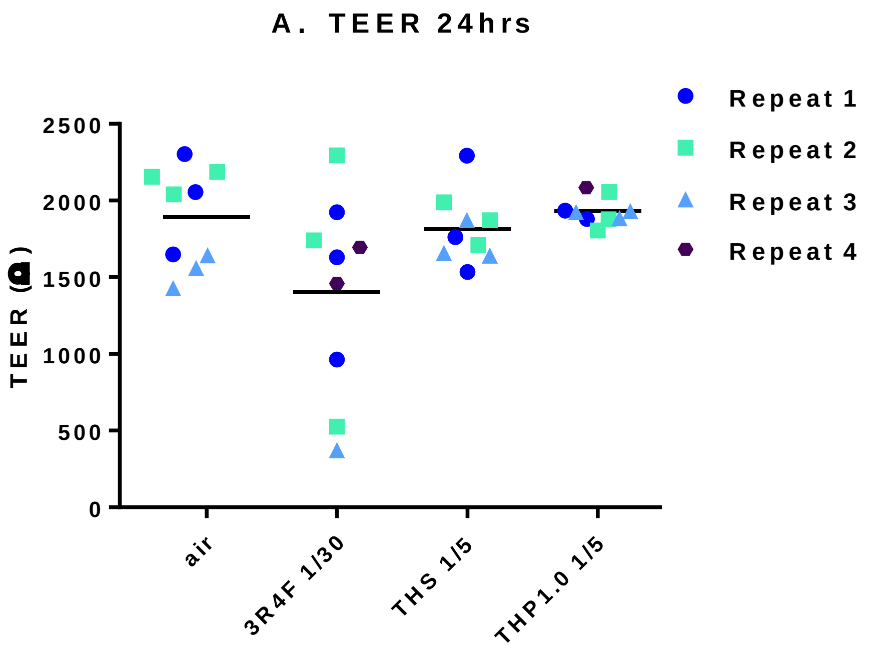


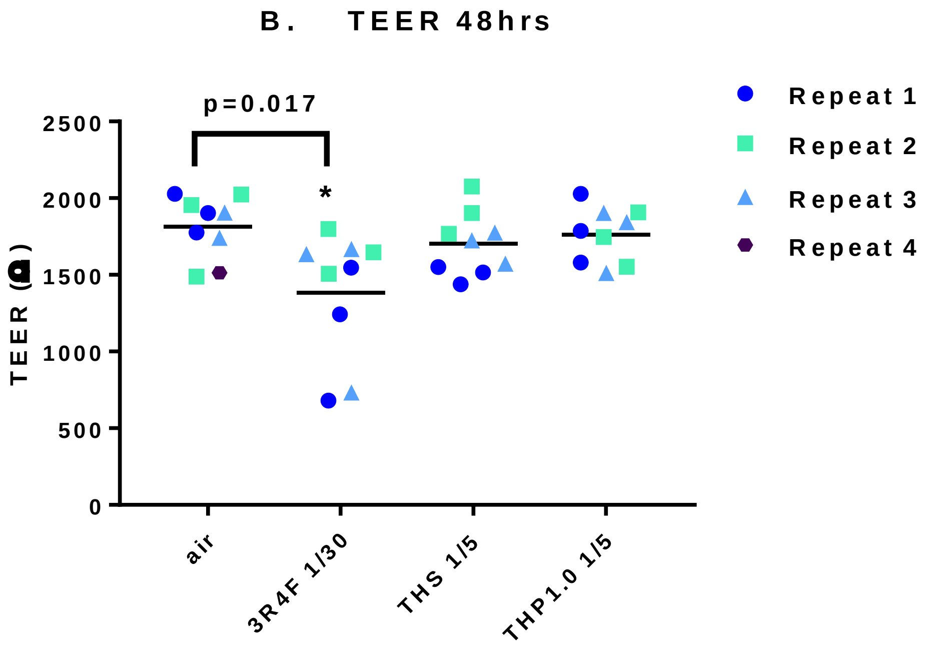

**Supplementary Figure S3:** Scatter plot of MucilAir™ percentage of LDH release after exposure to air, 3R4F smoke 1/30, THS 1/5 and THP1.0 1/5 aerosol. LDH at 24hrs (A) and 48hrs (B) post treatment. * denotes a significant difference versus air control at $p<0.05$.

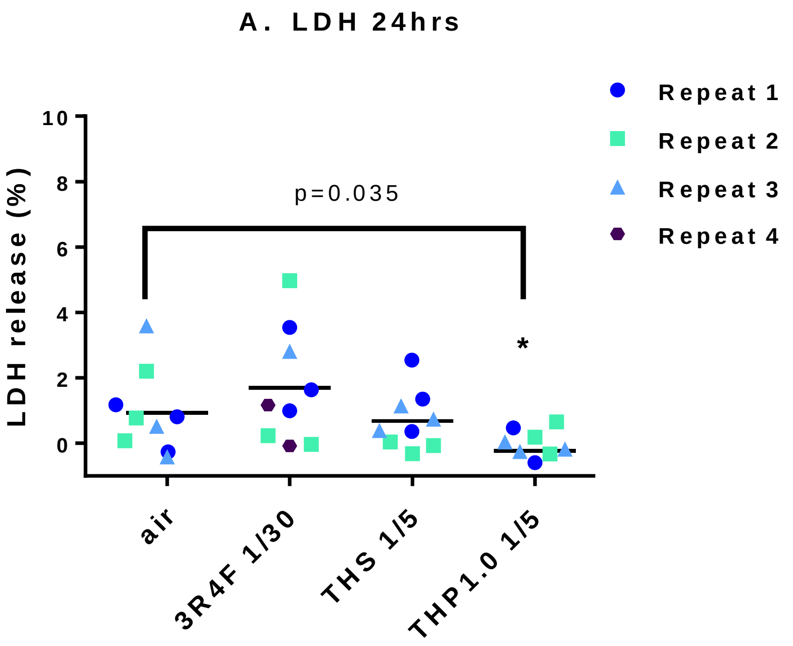


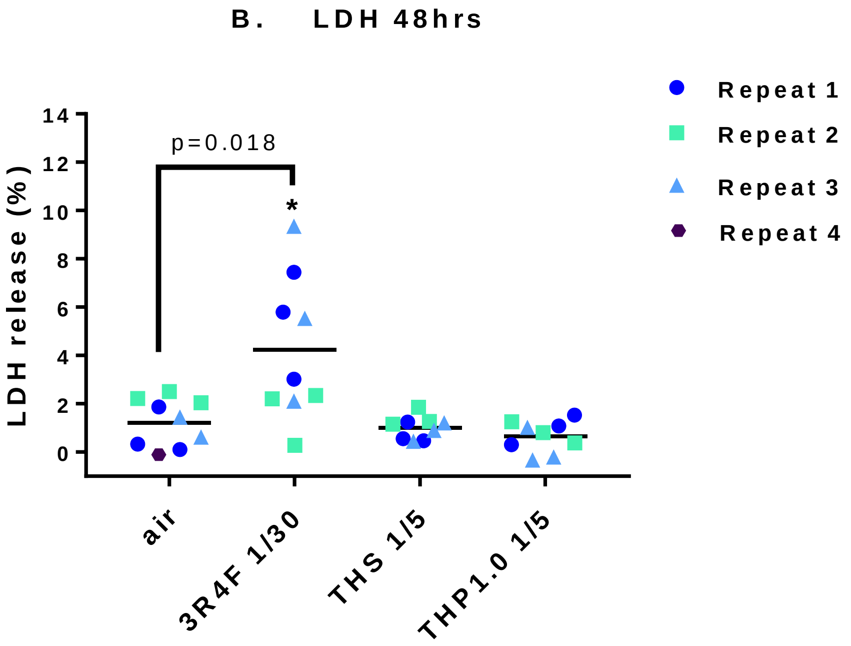

**Supplementary Figure S4:** Venn diagrams of significant genes (pFDR<0.05) differentially expressed (no fold change filter applied) in 3 donors after exposure to cigarette smoke from 3 different studies. Donor MD059401: this study, 3R4F cigarette smoke exposure vs air (raw sequence files: [https://www.ncbi.nlm.nih.gov/sra/SRP126155](https://www.ncbi.nlm.nih.gov/sra/SRP126155)), donor MD046001: unpublished study, 1R6F cigarette smoke exposure vs air (raw sequence files: [https://www.ncbi.nlm.nih.gov/sra/SRPSRP126705](https://www.ncbi.nlm.nih.gov/sra/SRPSRP126705)), and donor MD058501, 3R4F cigarette smoke exposure vs air (published study Haswell *et al*, 2017: raw sequence files: [https://www.ncbi.nlm.nih.gov/sra/SRP096285](https://www.ncbi.nlm.nih.gov/sra/SRP096285)). **A.** Venn diagram for up-regulated genes. **B.** Venn diagram for down-regulated genes.

**A.** **B.**

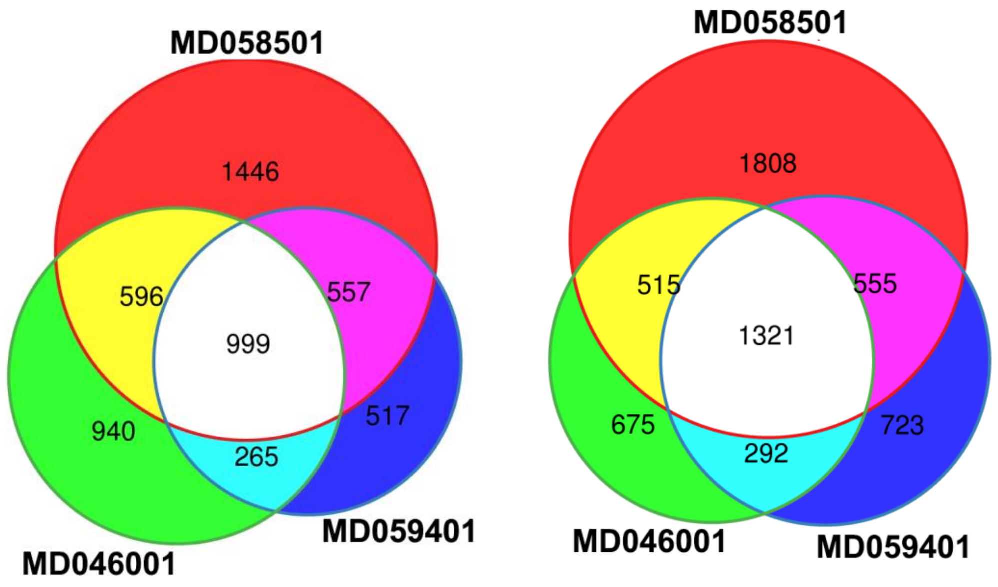

Supplement: Supplementary file 1 — Supplementary Figures S1 to S4 [file 41598_2018_19627_MOESM1_ESM.pdf]
